# Insights into the mechanisms governing P01 scorpion toxin effect against U87 glioblastoma cells oncogenesis

**DOI:** 10.3389/fphar.2023.1203247

**Published:** 2023-06-23

**Authors:** Saoussen Mlayah-Bellalouna, Dorra Aissaoui-Zid, Aurelie Chantome, Jed Jebali, Soumaya Souid, Emna Ayedi, Hafedh Mejdoub, Maya Belghazi, Naziha Marrakchi, Khadija Essafi-Benkhadir, Christophe Vandier, Najet Srairi-Abid

**Affiliations:** ^1^ LR20IPT01 Biomolécules, Venins et Application Théranostique, Institut Pasteur de Tunis, Université de Tunis El Manar, Tunis, Tunisia; ^2^ N2C UMR 1069, Institut national de la santé et de la recherche médicale, University of Tours, Tours, France; ^3^ LR16IPT04 Laboratoire d’Epidémiologie Moléculaire et Pathologie Expérimentale, Institut Pasteur de Tunis, Université de Tunis El Manar, Tunis, Tunisia; ^4^ USCR Séquenceur de Protéines, Faculté des Sciences de Sfax, Route de Soukra, Sfax, Tunisia; ^5^ Aix Marseille Université, CNRS, Plateforme Protéomique, IMM FR3479, Marseille Protéomique (MaP), Marseille, France

**Keywords:** small conductance calcium activated potassium channel, SK2 channel subtype, glioblastoma, androctonus australis scorpion venom, P01 toxin

## Abstract

The emerging concept of small conductance Ca^2+^-activated potassium channels (SK_Ca_) as pharmacological target for cancer treatment has significantly increased in recent years. In this study, we isolated the P01 toxin from *Androctonus australis (Aa)* scorpion venom and investigated its effect on biological properties of glioblastoma U87, breast MDA-MB231 and colon adenocarcinoma LS174 cancer cell lines. Our results showed that P01 was active only on U87 glioblastoma cells. It inhibited their proliferation, adhesion and migration with IC_50_ values in the micromolar range. We have also shown that P01 reduced the amplitude of the currents recorded in HEK293 cells expressing SK2 channels with an IC_50_ value of 3 pM, while it had no effect on those expressing SK3 channels. The investigation of the SK_Ca_ channels expression pattern showed that SK2 transcripts were expressed differently in the three cancer cell lines. Particularly, we highlighted the presence of SK2 isoforms in U87 cells, which could explain and rely on the specific activity of P01 on this cell line. These experimental data highlighted the usefulness of scorpion peptides to decipher the role of SK_Ca_ channels in the tumorigenesis process, and develop potential therapeutic molecules targeting glioblastoma with high selectivity.

## 1 Introduction

Potassium (K^+^) channels are the most diverse class of ion channels and are widely distributed in a variety of cells including cancer cells, where they are implicated in different stages of their development (Srairi-Abid et al., 2019). K^+^ channel family described in tumor cells, includes Ca^2+^-activated K^+^ channels (K_Ca_), Shaker-type voltage-gated K^+^ channels, ether-a-go-go (EAG) K^+^ channels, inward rectifier K^+^ channels, ATP sensitive K^+^ channels and swelling-activated K^+^ channels (Wang, 2004). Particularly, the expression of K_Ca_ channels, including small-conductance K_Ca_ (SK_Ca_) channels (consisting of SK1 (K_Ca_2.1), SK2 (K_Ca_2.2), and SK3 (K_Ca_2.3) subtypes), intermediate-conductance (IK_Ca_/SK4) and big-conductance (BKCa) channels in tumor cells has gained interest in the cancer field. They are associated with cell cycle progression, migration/invasion, cell volume control and apoptosis (Prevarskaya et al., 2010). SKCa channels are expressed in several cancer cell types and have been reported to be implicated in processes related to tumor cell survival (Krabbendam et al., 2020). Indeed, SK3 channels play a predominant role in melanoma and breast cancer cell migration (Potier et al., 2006; Chantome et al., 2009; Girault et al., 2012). On the other hand, SK4 channels have been involved in the migration potency of glioblastoma stem cells (Ruggieri et al., 2012). Thus, SKCa channels could be considered as biomarkers for carcinogenesis diagnosis and pharmacological targets for cancer treatment.

In this context, scorpion venom was shown to contain toxins and peptides which are specific blockers of these channels. Recently, the anticancer activities of some natural peptides/toxins attracted considerable attention in drug discovery and have been the object of several studies showing that they affect tumor growth, induce apoptosis and inhibit cancer metastasis and angiogenesis *in vitro* and *in vivo* (Ding et al., 2014; Majc et al., 2022). Several examples of the activity of these peptides/toxins as well as their effect on K^+^ channels have been recently summarized in many reviews. These peptides/toxins allowed highlighting the implication of different ion channels in cancerogenesis (Srairi-Abid et al., 2019). For instance, charybdotoxin (from *Leiurus quinquestriatus*), a known blocker of IK_Ca_/SK4, Kv1.3, and BK_Ca_ channels, inhibited proliferation and cell cycle progression in pancreatic and endometrial cancer cell lines (Wang, 2004). Moreover, tapamin, a toxin isolated from the *Mesobuthus tamulus* scorpion, blocked some cancer-related ion channels, such as SK_Ca_ and IK_Ca_/SK4, and exerts a cytotoxic effect (Díaz-García et al., 2020).

In this work, we isolated and purified P01 from *Androctonus australis* (Aa) venom, previously reported as inhibiting binding of ^125^I-apamin, a selective blocker of SK_Ca_ channels. P01 was used for studying its effects on the proliferation, migration and adhesion of three human cancer cell lines: U87 cells, derived from glioblastoma; MDA-MB231 cells, from breast cancer; and LS174 cells, from colon adenocarcinoma. Electrophysiological recordings, QPCR and Western blot studies were undertaken to highlight the involvement of SK2 channels in U87 glioblastoma tumorigenesis.

## 2 Experimental procedures

### 2.1 Scorpion venom and reagents

Venom of *Androctonus australis* scorpion from Beni Khedach (Tunisia) was collected by the veterinarian service of the Pasteur Institute of Tunisia (IPT) and kept frozen at −20^°^C in its crude form until use.

Chemicals (reagent grade) were purchased from Sigma Chemical Company, unless indicated otherwise.

Cell culture supplements and reagents were purchased from GIBCO (Cergy-Pontoise, France). Extracellular matrices were purchased from Sigma (St Louis, MO).

### 2.2 Cells lines and cellular culture

The ATCC (American Type Culture Collection, Rockville, MD, United States) cancer cell lines U87 (glioblastoma), MDA-MB231 (breast cancer) and LS174 (colon adenocarcinoma) were routinely cultured in DMEM supplemented with 10% fœtal bovine serum (FBS), 1% l-glutamine and 100 IU/mL penicillin/streptomycin. The fetal kidney HEK293 and HEK293T cell lines that do not express native SK_Ca_ channels were used for transfection experiments with plasmids encoding for the rat SK2 and human SK3. Rat SK2 cDNA cloned into pJA5 plasmid was a gift from Dr. Skolnik (university Langone Medical center, New York). Human SK3 cDNA cloned into Pipru vector was a king gift from Pr. Soriani (Université Côte d’Azur, CNRS, Inserm, Nice, France).

HEK293T-hSK3 and HEK293-rSK2 cells were selected using the puromycin and neomycin resistance gene respectively. HEK cells were routinely cultured in DMEM supplemented with 5% FBS and 1% l-glutamine. All cell lines were maintained at 37°C in a humid atmosphere of 5% CO_2_ in air.

### 2.3 Purification of the scorpion venom

Crude venom was extracted with cold water (1:4 v/v), then centrifuged at 15,000 *g* for 15 min. The supernatant was loaded on Sephadex G-50 gel filtration chromatography column (K26/100) equilibrated with 0.1 M acetic acid as described by (Miranda et al., 1970). The major fraction was lyophilized and fractionated by FPLC (Dionex Ultimate 3,000, Germany). A Resource S column (HR 5/5, 6 mL 16 × 30 mm, GE Helthcare, Sweden) pre-equilibrated with 0.05 M ammonium acetate (pH 6.6) was used. Proteins were eluted with a 40 min linear gradient from 0.05 to 0.5 M ammonium acetate, (pH 6.6) at a flow rate of 0.8 mL/min. Absorbance was monitored at 280 nm, Elution was controlled by the software Chromeleon (version 6.80). High Performance Liquid Chromatography (HPLC) purification of the Non Retained (NR) AaG50 fraction (Srairi-Abid et al., 2005), was performed on a C18 reversed-phase HPLC column (5 µm 4.6 × 250 mm, Beckman Fullerton, CA, United States). The elution and detection were performed by Beckman Coulter Series125 pump and a Beckman diode array detector set, respectively and controlled by means of the 32 Karat software (Beckman Coulter Fulletrton CA United States). Proteins were eluted from the column at a flow rate of 0.8 mL/min, using a linear gradient (45 min) from 15% to 45% of buffer B (0.1% TFA in CH_3_CN) in buffer A (0.1% TFA in water). The polypeptide concentration was determined using QuantiPro BCA Assay Kit (Sigma Aldrich).

### 2.4 Mass spectrometry and amino acid sequence determination

Samples were analyzed on an Ettan MALDI-Tof Pro (GE Healthcare Uppsala, Sweden) operating in positive reflectron mode with delayed extraction. The sample was co-crystallized with a 5 mg/mL solution of-cyano-4-hydroxy cinnamic acid (HCCA) on the MALDI target by the dry droplet method. MALDI spectra were acquired with an accelerating potential of 20 kV and a laser power set to the minimum level necessary to get a good signal. Mass calibration of the spectra was based on external calibration using appropriate peptide standards (Pepmix4, Laserbiolabs, Nice, France). Spectra acquired were analyzed on MoverZ software (Genomic Solutions,United States).

Reduction of 2 µg peptide with dithiothreitol and alkylation with 4-vinylpyridine, were performed as previously described (Srairi-Abid et al., 2000). The amino acid sequence was compared with those deposited in NCBI database, using FASTA (https://www.ebi.ac.uk/Tools/sss/fasta/) and BLAST program (https://blast.ncbi.nlm.nih.gov/Blast.cgi?PAGE=Proteins). Alignment was performed using CLUSTALW2 (https://www.ebi.ac.uk/Tools/msa/clustalw2/).

### 2.5 Cell viability assay

The human cell lines U87, MDA-MB231, or LS174 (10,000 cells per well) were treated with P01 peptide at different concentrations (25; 50; 100 and 200 μg/mL). After 24 h, the wells were washed with phosphate-buffered saline (PBS) and then the 3-(4,5-dimethylthiazol-2-yl)-2,5-diphenyltetrazolium bromide (MTT) (0.5 mg/mL) was added to the cells as previously described (Kouba et al., 2020). The crystals formed after the reduction of MTT by mitochondrial dehydrogenases were dissolved with dimethylsulfoxyde (DMSO). The quantification of live cells was achieved by measuring absorbance at 560 nm using a spectrophotometer (Thermo-Multiskan EX, Shanghai, China).

### 2.6 Cell proliferation test

The effect of the P01 peptide on cell proliferation was studied. The human cell lines U87, MDA-MB231 or LS174 (5,000 cells per well) were treated with 2; 5 and 10 μg/mL of P01 peptide. The plates were placed at 37°C for 72 h. Cellular proliferation was quantified at 560 nm by a spectrophotometer (Thermo-Multiskan EX, Shanghai, China) after treatment with 1% glutaraldehyde and staining with 0.1% crystal violet.

Every 24 h, a series of wells was washed with PBS, the cells were fixed with 1% glutaraldehyde, and preserved in PBS to follow the kinetics of proliferation. After 72 h, the cells were stained with crystal violet 0.1% and quantified by measuring the absorbance at 560 nm using a spectrophotometer (Thermo-Multiskan EX, Shanghai, China).

### 2.7 Cell migration assay

Cell migration assay was performed using modified Boyden chamber (NeuroProbe Inc., Bethesda, MD, United States) as previously described (Sarray et al., 2007). Membranes were coated with fibrinogen (Fg) (50 μg/mL), fibronectin (Fn) (10 μg/mL), collagen type I (Coll-I) (50 μg/mL) for U87, MDA-MB231 and LS174 cells respectively, for 2 h at 37°C. Cells were harvested as a single cell suspension (10^6^ cells/mL) and treated with P01 at 50 μg/mL. Then added to pre-coated membranes and allowed to migrate for 5 h at 37°C in Boyden chamber. Cells were fixed on the underside of the membrane, stained by 0.1% crystal violet and migration was quantified by measuring the absorbance at 560 nm using a spectrophotometer (Thermo-Multiskan EX, Shanghai, China).

### 2.8 Cell adhesion assay

Adhesion assays were performed as previously described by (Sarray et al., 2007). Glioblastoma U87 cells in suspension (10^6^ cells/mL) were treated with 50 μg/mL of P01 and deposited on wells coated with purified extracellular matrix (ECM): Fg at 50 μg/mL; Fn at 10 μg/mL; Coll-I at 50 μg/mL; laminin (Lam) at 15 μg/mL or poly-L-lysine (PL) at 20 μg/mL for U87. MDA-MB231 and LS174 cells were also used in their adequate ECM: Fg and Coll-I respectively. They were incubated to adhere to the substrate for 2 h or 24 h at 37°C. After washing, attached cells were fixed, stained by 0.1% crystal violet, lysed with 1% SDS and quantified by measuring absorbance at 560 nm. The specific adhesion is obtained by subtracting the absorbance obtained in the absence of adhesion substrate (no specific control). To study the dose-response effect, different concentrations of P01 were used (25–100 μg/mL) and the IC_50_ value was determined.

To study the implication of integrins in the P01 peptide activity, adhesion assays using anti-integrin blocking antibodies: anti- α1β1 (1/200), anti- αvβ3 (1/200), anti- α5 (1/200) and anti- β3 (1/200) were performed as described above, except that cells were pretreated with the active peptide P01 at 50 μg/mL.

### 2.9 PCR analysis

#### 2.9.1 Total RNA extraction

RNA was extracted as previously described by (Jebali et al., 2014). Cells (1 × 10^6^) were washed in ice-cold PBS and lysed in the Trizol buffer (Invitrogen) according to the manufacturer’s instructions. The supernatant was cleared by centrifugation, Isopropanol-precipitated and resuspended in sterile Diethylpyrocarbonate (DEPC) 0.1%: nuclease inhibitor-water (Amersham-Pharmacia).

#### 2.9.2 Reverse transcription (RT-PCR) and PCR amplification

Reverse transcription of total RNA was operated using the transcriptase reverse M-MLV RT (Moloney Murine Leukemia Virus-Reverse Transcriptase) according to the manufacturer’s instructions. The reverse transcription reaction was performed with a mixture containing: 1 µL RNA (10 ng), 4 µL of First strand buffer (5X buffer) (Amersham-Pharmacia), 1 µL of dNTP (1 mM), 2 µL of oligo d (T) (0.1 mg/mL), 2 µL of DTT (100 mM) and 8.5 µL of DEPC H_2_O for a final volume of 20 µL. The samples were mixed and incubated for 5 min at 65°C, then after a short centrifugation, a second incubation of 10 min at 37°C was performed. 1 μL of reverse transcriptase M-MLV RT (200 IU) and 0.5 µL of RNase inhibitor (RNasin (Ribonuclease inhibitor 40 IU) were then added and incubated for 1 h at 37°C. The inactivation of the reverse transcriptase is carried out for 5 min at 95°C.

The PCR reaction was performed in a final volume of 50 µL of the reaction mixture containing 0.5 µg of cDNA; 20 picomoles of each of the two primers corresponding at both ends of the target DNA (Table 1); 10 mM of each dNTP; 1x Taq buffer (50 mM KCl, 10 mM pH 8.3 and 1.5 mM MgCl_2_) and 1 units of Taq polymerase (Amersham-Pharmacia). The PCR protocol included an initial denaturation step at 95°C for 5 min followed by 35 cycles of denaturation (30 s at 94°C), annealing (30 s at 55°C) and extension (30 s at 72°C), and a final extension for 7 min at 72°C. The amplified fragments were separated on 1% agarose gel electrophoresis. The Amplicon was visualized under UV light on a transilluminator to be photographed.

**TABLE 1:** The primer sequences used for PCR and qPCR reactions.

Primer	Sequence	Amplicon size (bp)
F-hGAPDH	5′CGC​TCT​CTG​CTC​CTC​CTG​TT 3′	310
R-hGAPDH	3′CCA​TGG​TGT​CTG​AGC​GAT​GT 5′	
F-SK1	5′AGG​GAG​ACG​TGG​CTC​ATC​TA3′	158
R-SK1	3′TTA​GCC​TGG​TCG​TTC​AGC​TT5′	
F-SK2	5′GAC​TTG​GCA​AAG​ACC​CAG​AA	230
R-SK2	3′CCG​CTC​AGC​ATT​GTA​AGT​GA5′	
F-SK3	5′GTT​TGG​AAT​TGT​TGT​TAT​GGT​GA3′	130
R-SK3	3′GAT​GAT​CAA​GCC​CAA​AAG​GA5′	

#### 2.9.3 Quantitative-PCR

One-step quantitative real-time reverse transcription polymerase chain reaction (Q-PCR) was performed on a LightCycler^®^480 II System from Roche using the LightCycler480 SW 1.5 Software. Brilliant II SYBR_Green QPCR Master Mix was used for the Q-PCR reaction in a final volume of 25 µL of the reaction mixture. The experimental reaction was prepared as recommended by the manufacturer. The primer sequences used are presented in Table 1. The endogen control GAPDH was used as a housekeeping gene and was prepared in the same conditions. The alternative protocol with Three-Step Cycling, cited in the manufacturer instructions, was used with an initial denaturation step of 10 min at 95°C followed by 40 cycles. Each cycle comprises 3 steps: DNA denaturation at 95°C for 30 s, a specific hybridization of the SK1, SK2 and SK3 primers for 1 min at 58°C and a polymerization at 72°C for 30 s. Primer pair specificity was tested by amplification of the target using 10 ng of DNA.

### 2.10 Western blot analysis

Protein expression of the SK2 channel was assessed by Western blotting analysis using a polyclonal antibody raised against synthetic peptide ETQMENYDKHVTYNAERS corresponding to a region of SK2 (Clinisciences). U87, LS174 and MDA-MB-231 cells were plated at a density of 3.10^5^ cells/well in 6-well culture plates in DMEM medium supplemented with 10% FBS (Fetal Bovine Serum) and allowed to adhere overnight. Cells were washed twice with PBS and subsequently lysed with Laemmli buffer (1X) at room temperature. About 50 µg of whole cell extracts were resolved in SDS-polyacrylamide gels (10%) and transferred onto a polyvinylidene difluoride (PVDF) membrane (Immobilon-P, Millipore). The immune-reactive proteins were visualized by the enhanced chemiluminescence detection system (ECL, Pierce, Rockford).

### 2.11 Patch-clamp experiments

Experiments were performed with cells seeded into 35-mm Petri dishes at 3,000 to 6,000 cells per cm^2^. All experiments were performed using the conventional whole-cell recording configuration of the patch-clamp technique (Girault et al., 2011). For HEK cells expressing SK2 or SK3 channels, voltages clamp ramp protocols were used with a duration of 2 s, with varying voltages - 100 to +100 mV, with a holding potential of 0 mV. This protocol is suitable for studying the channels whose activation (and inactivation) is independent of the voltage and the time, which is the case of SK_Ca_ channels. The compositions of extracellular solution (in mM: NaCl 140, KCl 4, MgCl_2_ 1, NaH_2_PO_4_ 0.33, CaCl_2_2 HEPES 10, and D-glucose 11.5) and the intracellular pipette solution (KCl 145, MgCl_2_ 1, Mg-ATP 1, CaCl_2_ 0.87, EGTA 1, HEPES 10; the pCa is of 6 (10^−6^ M of calcium concentration) are such that predominantly visible currents, resulting from this imposed voltage protocol, are due to the activity of K^+^ channels, particularly SK_Ca_. Indeed, using these solutions, the equilibrium potential for chlorine (E_Cl_-) in our conditions is zero, due to the presence of an identical amount of chloride ions on each side of the cell membrane (E_Cl-_ = 0 mV). To measure the amplitude of the currents in HEK cells overexpressing channels of interest, we positioned ourselves to this membrane voltage (Vm = 0 mV). Also, at 0 mV, the membrane potential currents will be recorded mainly K^+^ currents and not Cl^−^ currents.

For U87 cells, whole-cell K^+^ currents were generated by stepwise 8 mV depolarizing pulses from a constant holding potential of −90 up to +60 mV. The composition of extracellular solution is the same as that described above and the intracellular pipette solution is as follows in mM: K-glutamate 125, KCl 20, MgCl_2_ 1, Mg-ATP 1, HEPES 10, CaCl_2_ 0.7, EGTA 1, and pH was adjusted to 7.2 with KOH. The pCa is of 6.4 (10^−6.4^ M of calcium concentration). Leidab-7 (Tocris, France) was used as reference peptide to selectively block SK2 currents in U87 cells.

### 2.12 Statistics

Statistical analysis, evaluated by one-way analysis of variance (ANOVA) done with GraphPad Prism 6 (GraphPad Softweare, San Diego, CA, United States), was made using Student t test. Data are reported as mean ± SD. Statistical comparisons between three or more sets of data were performed. Differences were considered significant when *p* < 0.05.

## 3 Results

### 3.1 Purification and identification of P01

**FIGURE 1:**
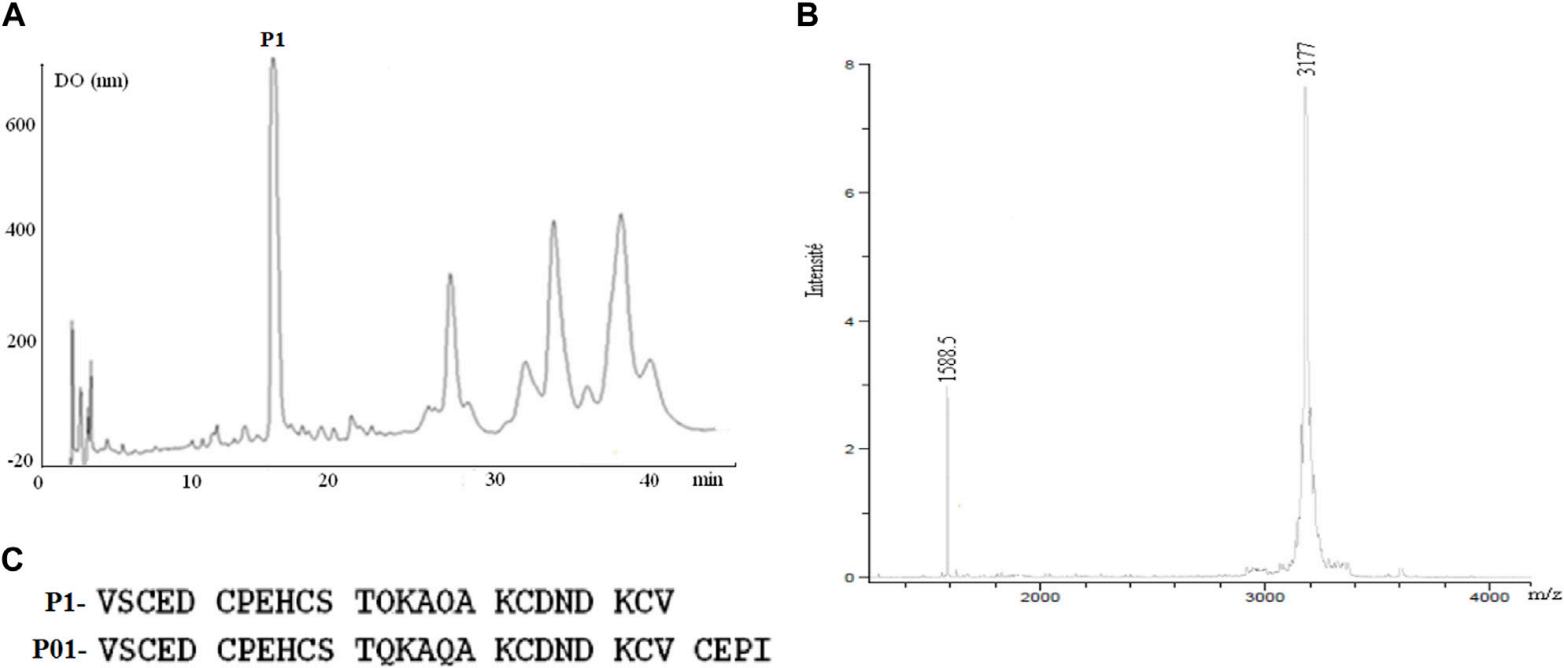
**(A)** Chromatogram of the HPLC purification of P01: C18 with a linear gradient from 15% to 45% buffer B (0.1% TFA in CH_3_CN) in A (water 0.1% TFA) at a flow rate of 0.8 mL/min over 45 min. **(B)** the major fraction 1 has a mass of 3,177 Da. **(C)** Comparison of Amino acid sequence obtained by the Edman method of fraction P1 and sequence of the P01 toxin (Zerrouk et al., 1996).

The toxic fraction AaHG50 representing 33% of the crude venom and containing peptides of 3–7 kDa (Miranda et al., 1970) was obtained by sephadex-G50 chromatography of Aa venom. FPLC of AahG50 has been performed as previously described by (Srairi-Abid et al., 2005). The non-retained AaG50 fraction was injected in an HPLC system using C18 column (Figure 1A). A mass spectrometry analysis of the first major peptide P1 gives a mass of 3,177 Da (Figure 1B).

The Edman degradation of 1 nmol of the native peptide allowed the identification of the 25 first amino acids of its sequence (Figure 1C). Comparison with protein sequences existing in the databases indicates that this peptide showed complete identity with the N-terminal sequence of P01 toxin (Figure 1C). Furthermore, fragmentation of our peptide and that of P01 (provided by Maya Belghazi), using ULTRAFLEX II mass spectrometer, showed a complete MSMS spectrum identity between the two peptides (data not shown).

### 3.2 P01 had a specific effect on U87 glioblastoma cells

**FIGURE 2:**
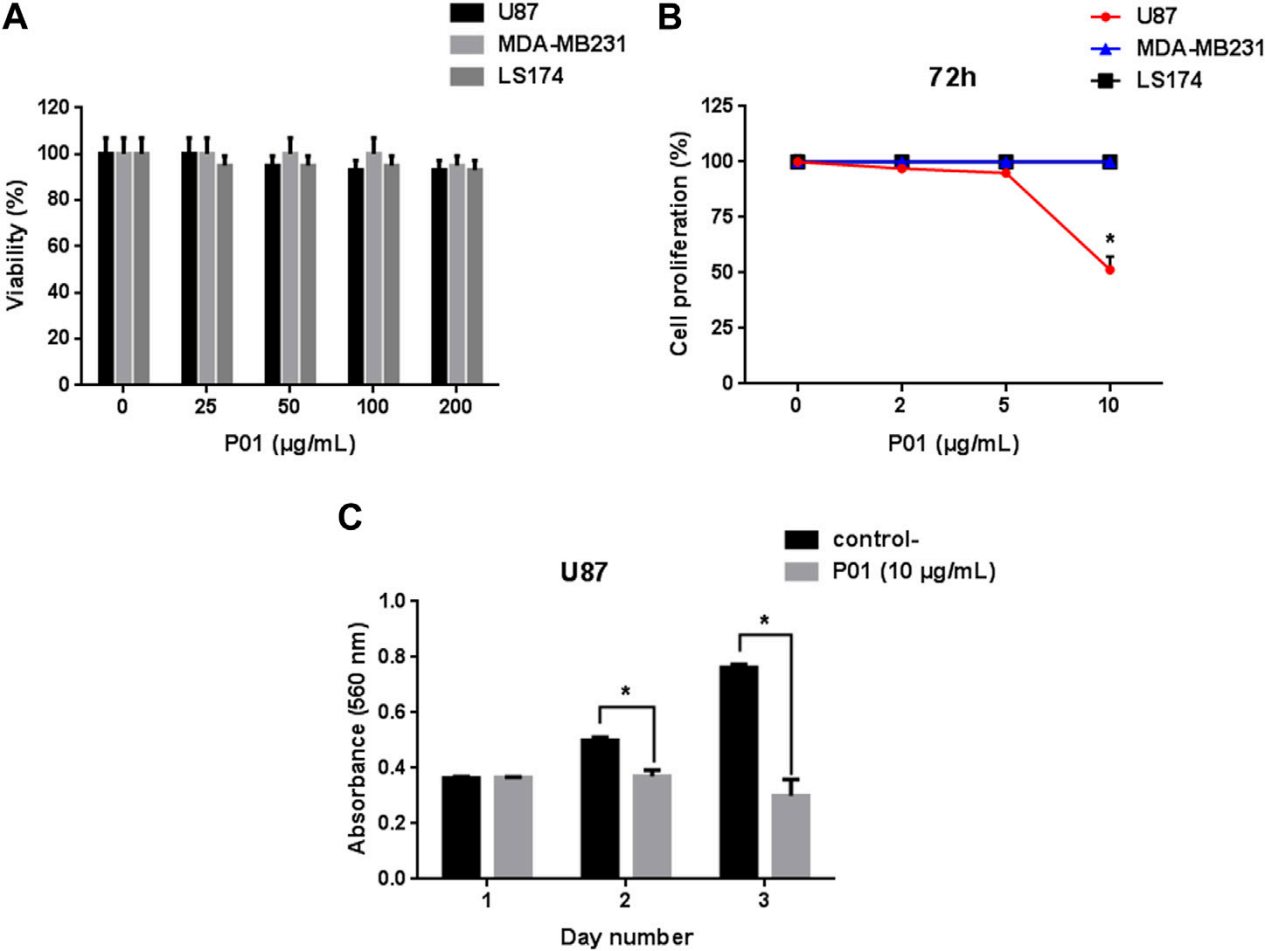
**(A)** Effect of P01 on the viability of cancer cell lines: U87, MDA-MB231, and LS174 cells were treated with 25–200 μg/mL of peptide for 24 h. **(B)** Dose-response effect of P01 on the proliferation of the three cancer cell lines after 72 h of incubation with 2–10 μg/mL of P01. **(C)** Kinetic effect of P01 at 10 μg/mL on U87 cell proliferation. The Mean, SD (*n* = 3). *p < 0.05, significantly different from control.

Cell viability was investigated on the three cancer cell lines U87, MDA-MB231 and LS174 using MTT assay. Our results showed that after 24 h of treatment, P01 at different concentrations (25–200 μg/mL) did not have a cytotoxic effect (Figure 2A) on the tested tumor cells that maintained a normal morphology. Interestingly, after 72 h, P01 at 10 μg/mL (3 µM) inhibited about 50% of U87 cell proliferation with no effect on MDA-MB231 and LS174 cells (Figure 2B). The kinetic effect of P01 on U87 cells showed that the inhibition appeared after 48 h of treatment (Figure 2C).

**FIGURE 3:**
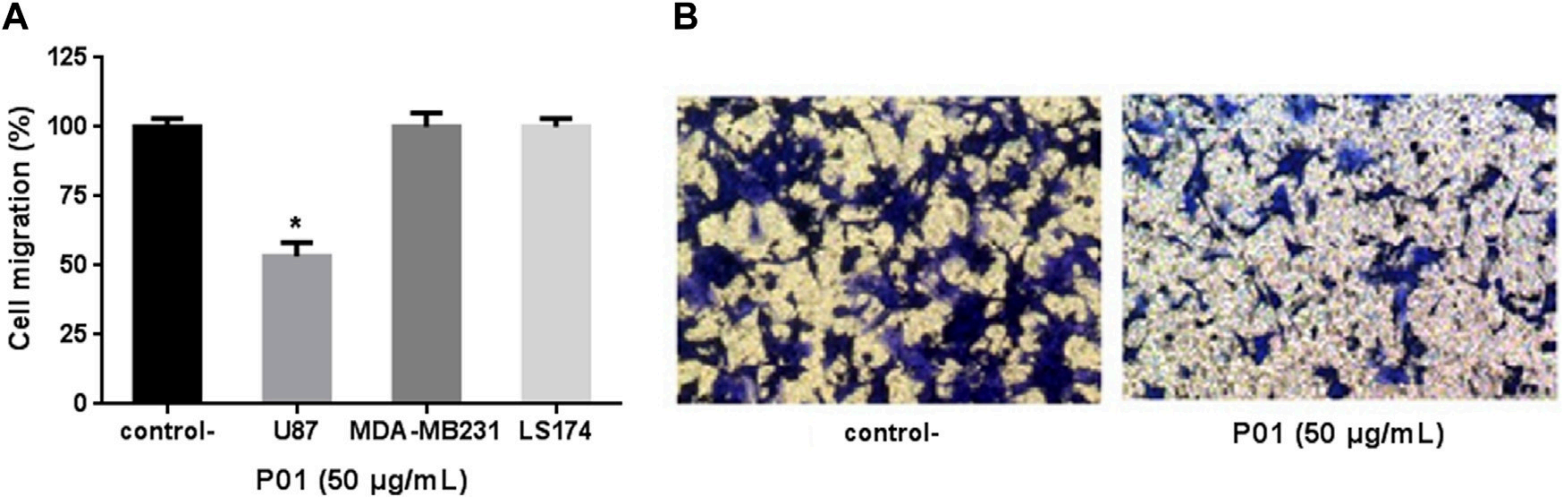
**(A)** Effect of P01 on U87, MDA-MB231, and LS174 cells migration. **(B)** Microscopic observation after migration, fixation and staining of U87 cells treated with 50 μg/mL of P01 compared to negative control. The Mean, SD (n = 3). *p < 0.05, significantly different from control.

When tested on the migration of the 3 cell lines, P01 (at 50 μg/mL) exhibited an anti-migratory effect only in U87 cells (48% of inhibition), while it had no effect neither on MDA-MB231 nor on LS174 cells (Figure 3).

**FIGURE 4:**
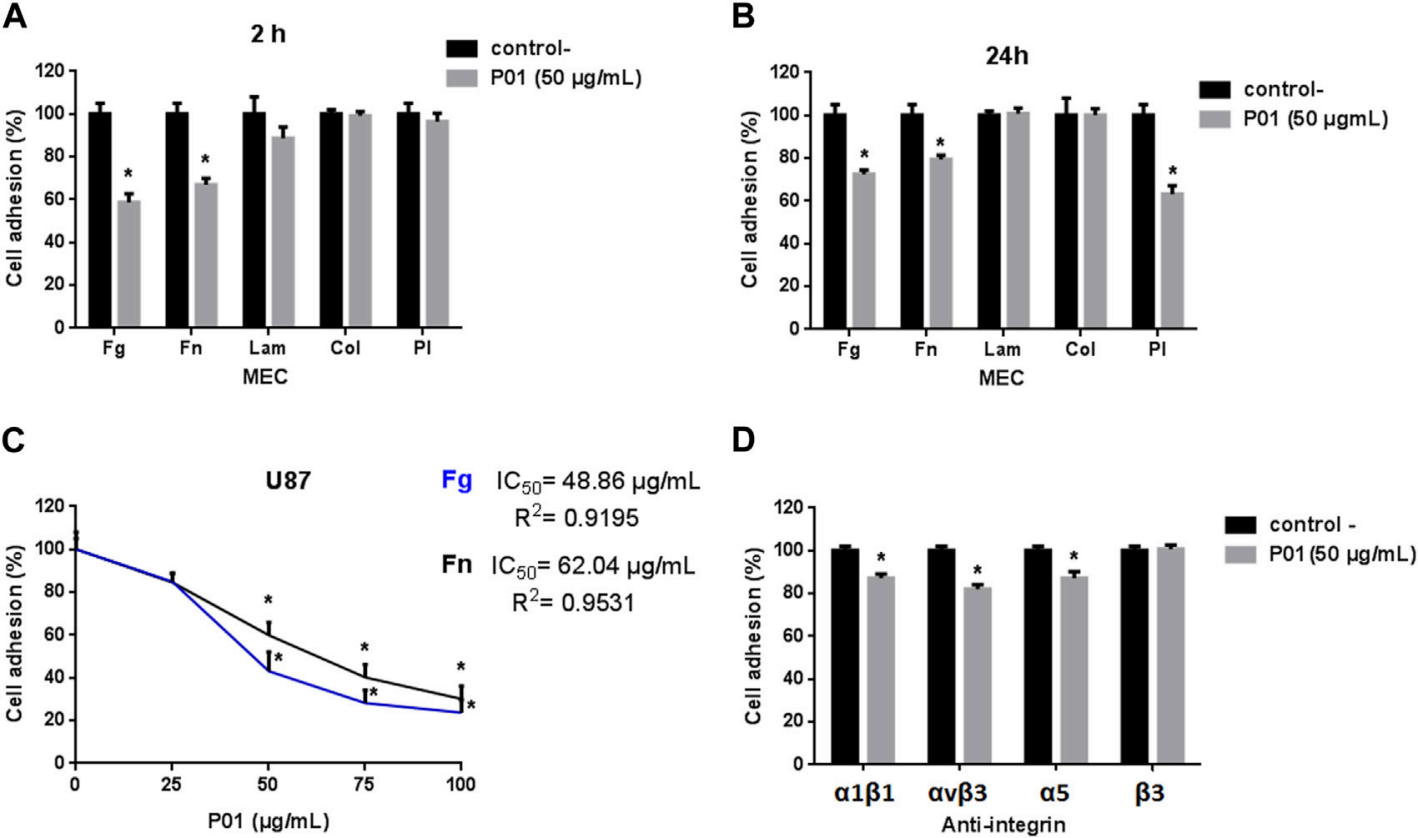
Effect of P01 on U87 cell adhesion **(A)** after 2 h, **(B)** after 24 h. Cells are treated with 50 μg/mL of P01 on Fg, Fn, Lam, Coll-I as ECM or Pl. **(C)** Dose-response effect of P01 on U87 cells adhesion to Fg and Fn, after 2 h of incubation. **(D)** Effect of P01 in the presence of anti-integrinic antibodies. Cells were pre-incubated in the absence (black bars) or in the presence (grey bars) of 50 μg/mL P01 for 30 min at room temperature and allowed to adhere for 2 h at 37°C in presence of anti-α1β1, -αvβ3, -α5, or -β3. The Mean, SD (*n* = 3). *p < 0.05, significantly different from control.

Likewise, P01 inhibited the adhesion of only U87 cells (data not shown). As shown in Figure 4A, after 2 h of incubation, the best inhibitory effects were obtained with Fg and Fn, as ECM, with a rate of 41% and 38%, respectively. Moreover, no inhibition could be observed on the integrin-independent substratum, poly-L-lysine (Pl), suggesting that the effect of P01 involves the integrins family as adhesion receptors. Based on these results, we studied the dose-response effect of P01 on these two ECM (Fg and Fn). The IC_50_ values of P01 were 48.86 μg/mL and 62.04 μg/mL, respectively (Figure 4C). After 24 h of incubation, P01 stayed active on Fg and Fn, and an inhibition effect on Pl appeared (Figure 4B).

To identify the possible targeted integrins, we checked the effect of P01 on the adhesion of U87 cells on some integrins linked to glioblastoma malignancy. As illustrated in Figure 4D, P01 was not able to alter cell adhesion through β3 integrins, but reduced the adhesive function of α1β1, αvβ3 and α5 integrins. Thus, our results highlighted that P01 had an anti-tumoral effect specifically on U87 glioblastoma cells and integrins could be associated with this activity.

### 3.3 SK2 channel subtype is expressed in the three cancer cell lines

Since P01 is a peptide ligand of the apamin-sensitive receptor (Zerrouk et al., 1996), it is thought that its activity could be *via* the inhibition of the SK_Ca_ channels. Accordingly, we have investigated the expression of SK channel subtypes in the three tested cancer cell lines.

**FIGURE 5:**
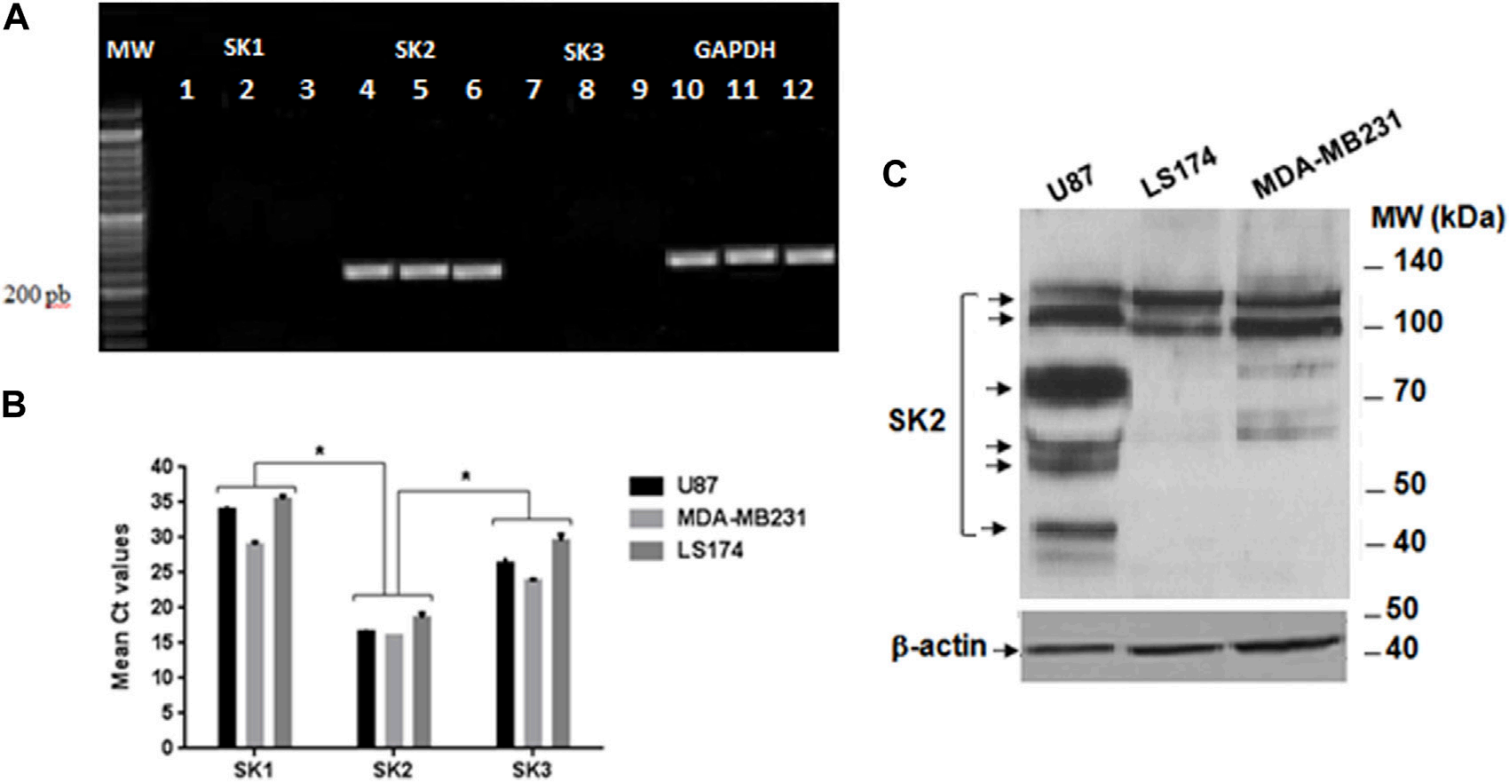
Expression of SK1, SK2, and SK3 channels on the three cancer cell lines. **(A)** Gel electrophoresis of the PCR products loaded on a BET agarose gel 1%. MW Molecular weight marker; Lanes 1, 2 et 3: expression of SK1 on U87, MDA-MB231 and LS72 cells lines respectively; Lane 4, 5, and 6: expression of SK2 on U87, MDA-MB231 and LS174 respectively; Lanes 7, 8, and 9 expression of SK3 on U87, MDA-MB231, and LS174; Lane 10, 11, and 12 expression of GADPH on U87, MDA-MB231, and LS174 respectively. **(B)** Cycle threshold (Ct) values of the SK1, SK2, and SK3 genes in U87, MDA-MB231, and LS174 cell lines, generated from the qPCR. **(C)** Western blot analysis with the specific SK2 antibody.

First, we carried out PCR analysis to verify the expression of SK_Ca_ channel subtypes genes in the three cancer cell lines. GAPDH (glyceraldehyde-3-phosphate deshydrogenase) was used as a housekeeping gene. The Figure 5A showed that only the amplified product of 230 bp, corresponding to the SK2 subtype of SK_Ca_ channels transcript, is expressed in the 3 cells lines with a Cycle threshold_(Ct) value of 16.84 ± 0.58, while neither SK1 (Ct = 27.335 ± 2.605) nor SK3 (Ct = 26 ± 0.36) expressions were noticed in the 3 cell lines (Figure 5B). Since the set of primers, used, allowed us to highlight the expression of SK2 channels in the three cancer cell lines, they are also exploited to quantify this channel by Q-RT-PCR.

Due to gene regulation at different levels, the expression of mRNA transcripts does not necessarily exhibit the same pattern of protein expression resulting in production of functional channels. Therefore, the SK2 protein expression in the three cancer cell lines was investigated by Western blot.

Our result showed that SK2 encodes several protein isoforms of molecular weight (MW) ranging from 40–50 kDa to 100–140 kDa and differentially expressed depending on the type of tumor cell line (Figure 5C). At a MW range from 100 to 140 kDa, while MDA-MB231 cells express two isoforms at the same level, U87 cells, in contrast, express high levels of a smaller one compared to LS174 cells showing increased expression of a larger molecular weight isoform (Figure 5C). Interestingly, four immuno positive protein bands (40–50 kDa; 50–70 kDa; 70–100 kDa) recognized by SK2 antibody were detected only in U87 cells (Figure 5C).

### 3.4 Effect of P01 on HEK cells expressing rSK2 and hSK3 channels

**FIGURE 6:**
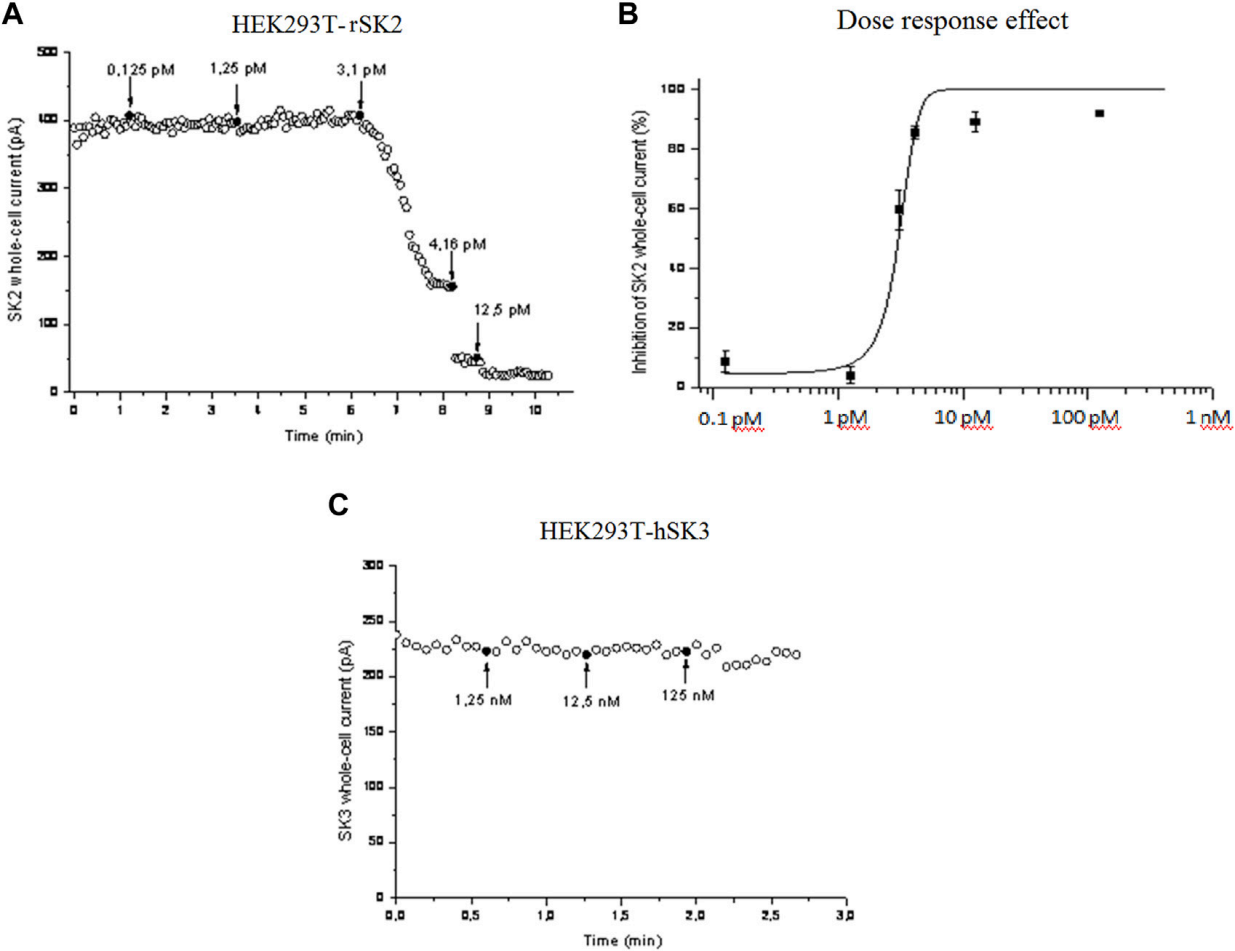
Effect of P01 on rat SK2 and human SK3 channels expressed in HEK293 cells. The inhibition of SK_Ca_ currents by P01 toxin were studied in dose-response experiments using whole-cell configuration of patch clamp (pCa 6). Voltages ramps were performed with a duration of 2 s, with varying voltages - 100 to +100 mV, with a holding potential of 0 mV. Amplitudes of SK2 **(A)** and Dose-response fit (*n* = 5) **(B)**. SK3 currents were recorded at 0 mV and plotted as a function of time. The bath solution was shifted to solutions with the indicated concentrations of P01 at the times marked by black arrows **(C)**. The solid curve was obtained after applying a Hill fitting procedure to the data-points.

We then checked whether P01 could affect the activity of SK2 channel compared to SK3 channel. Electrophysiological records by voltage-clamp showed that P01 reduced SK2 currents (Figure 6A). The SK2 currents reduction was dose-dependent giving an IC_50_ of 3 pM (Figure 6B), while no effect was noticed on the current of the SK3 channel (Figure 6C).

### 3.5 Effect of P01 on SK2 currents of U87 cells

**FIGURE 7:**
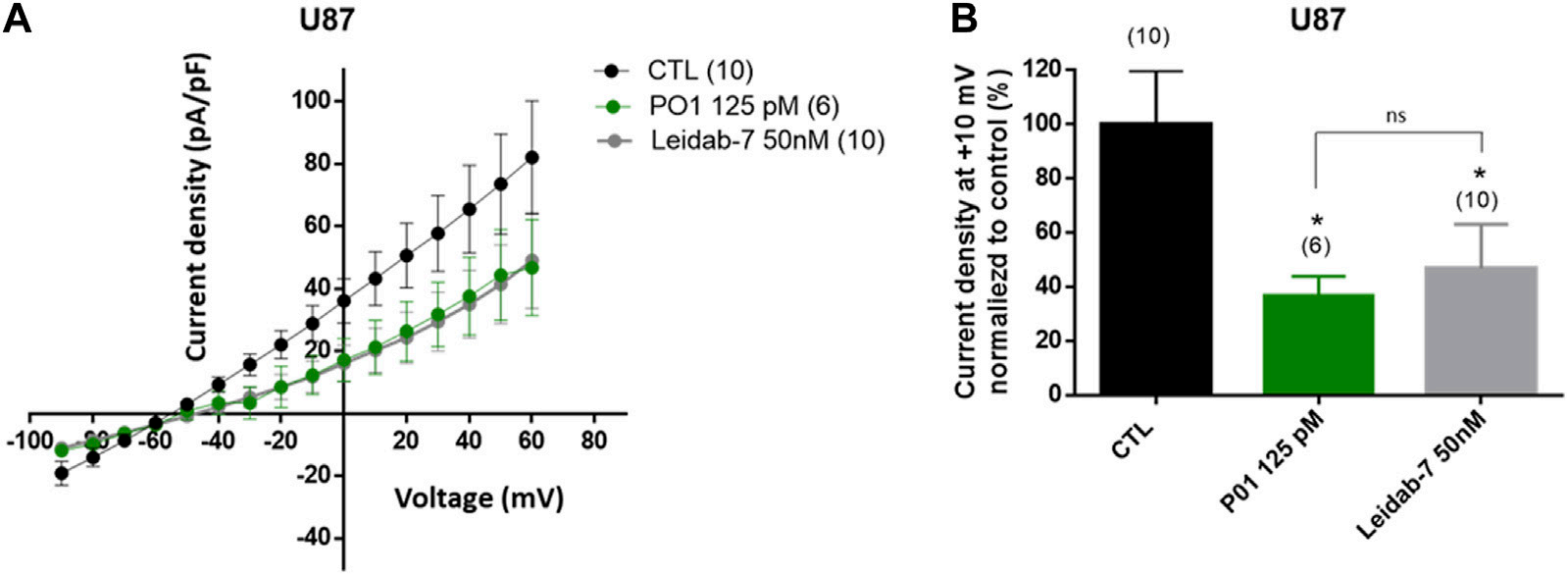
Effect of P01 on currents of U87 cells. The reduction of SK2 currents by P01 toxin were studied using whole-cell configuration of patch clamp (pCa6.5). Whole-cell currents were generated by stepwise 8 mV depolarizing pulses (400 ms duration; 5 s intervals) from a constant holding potential of −90 up to +60 mV. **(A)** Current density-voltage relationships obtained in control condition (CTL) and after application of P01 (125pM) or Leidab-7 (50 nM). Leidab-7 was used as reference peptide to selectively block SK2 currents. The current density–voltage relation was obtained by dividing the averaged steady-state currents elicited between −90 and +60 mV by the respective cell capacitance. **(B)** Current density at +10 mV normalized to control condition (%). Results represent the mean ± SEM. The Kruskal–Wallis test with Dunn’s correction was used. *: Significantly different from control condition at *p* < 0.05. ns: no significant different between P01 and Leidab-7 treatment on current density. The numbers in brackets indicate the number of cells.

Finally, we inspected if SK2 currents were detected in U87 cells. As shown Figure 7A, application of Leidab-7, a selective SK2 channel blocker caused a large reduction of the outward K^+^ currents compared to control condition (53% reduction at + 10 mV, Figure 7B) demonstrating the presence of SK2 channel conductance in the plasma membrane of U87 cells. As expected, current density-voltage relationships obtained with P01-treated cells and Leidab-7 treated cells were similar (Figures 7A, B) suggesting that P01 reduced endogenous SK2 conductance.

## 4 Discussion

Potassium channels have been implicated in many diseases, either in a primary etiologic role in channelopathies or as mediators in other pathogenesis. It is not surprising that roughly one third of all drugs used in modern therapies are ion channel modulators (Wickenden, 2002; Huang and Jan, 2014).

Fundamental studies have been accumulating evidence that tumor cells possess various types of K^+^ channels playing important roles in regulating their development, increasing evidence that cancer constitutes a category of channelopathies. The K^+^ channels involved in oncological processes belong to four main classes (Huang and Jan, 2014). Among these, KCa channels have gained interest in the cancer field. Indeed, they are associated with cell cycle progression, migration/invasion, cell volume control and apoptosis (Jäger et al., 2004; Wang, 2004). Interestingly, scorpion peptides, especially those blocking K^+^ channels have been investigated, as they represent specific ligands, and shown promising anticancer effects, although purification and characterization of active components still remain a challenge for novel cancer therapies (Ding et al., 2014).

Our team has initiated the fractionation of *Aa* scorpion venom, in order to identify scorpion peptides targeting potassium channels involved in the oncological process. Thus, we succeeded to isolate one peptide well represented in this venom. Based on its N-terminal sequence and its mass fingerprint this peptide corresponded to the P01 toxin, previously described by (Zerrouk et al., 1996). P01, classified as a α-Ktx8.1 toxin, was reported to inhibit the binding of ^125^I-apamin, a toxin from *Apis mellifera* bee venom that specifically blocked SK channel subtypes (Wei et al., 2005; Pedarzani and Stocker, 2008). In this work, we found that P01 exhibited a high inhibiting effect on the SK2 channel subtype expressed in HEK293 cell**s**, with an IC_50_ value of 3 pM, whereas it has no effect on the SK3 channel subtype. P01 appeared to be more active than apamin, which blocked hSK1 and rSK2 channels in HEK 293 cells with IC_50_ values of 3.3 nM and 83 pM, respectively (Strobaek et al., 2000). P01 has also a higher effect than leiurotoxin I (also called scyllatoxin), the first toxin isolated from *Leiurus quintestriatus* scorpin, which blocked the rSK2, channels, expressed in HEK293 cells with an IC_50_ value of 0.28 nM (Hosseini et al., 2001).

It is worthy to note that the effects of P01 on SK_Ca_ channel subtypes, recorded in our work, were different from those obtained by Shakkottai (Shakkottai et al., 2001), which showed that P01 had little or no blocking activity on SK2 or SK3 expressed on Jurkat cells. This can be explained by the differential expression of either functional SK2 isoforms or other membrane receptors between HEK293 and Jurkat cells. Indeed, in their work Shah and Haylett (Shah and Haylett, 2000) showed that apamin blocked hSK1 expressed in HEK293 and COS-7 cells with IC_50_ values of 8 nM and 12 nM, respectively, which are also different from that recorded on hSK1 channels expressed in *Xenopus oocytes* (100 pM) (Shah and Haylett, 2000). Accordingly, they demonstrate that the properties of the channel may depend on the expression system.

This can also explain the specific effect of P01 on U87 cells. Indeed, we found that P01 inhibited the viability of only U87 cells after 72 h of incubation with IC_50_ of 10 μg/mL (3.14 µM) and had no effect on those of MDA-MB231 and LS174 cells. The effect of P01 is nearly 2.5 times more important than that of KAaH2, a scorpion peptide isolated from the same venom (Srairi-Abid et al., 2005; Aissaoui et al., 2018). In fact, like P01, KAaH2 has an inhibiting effect on U87 cells proliferation, with an IC_50_ value of 8 μM, without being active neither on MDA-MB231 nor on LS174 cells (Aissaoui et al., 2018). In fact P01 could be considered as a potent peptide, comparing to peptides from scorpion venoms. Indeed it is also 6 times more active than the tetrapeptide AaTs1 isolated from the same venom (Aissaoui-Zid et al., 2021). This later inhibited the proliferation of U87 cells with an IC_50_ of 0.5 mM by up regulating the p53 and FPRL-1 expression.

When tested on cell adhesion and migration, as key processes of metastasis, P01 showed also a specific effect on U87 cells whereas MDA-MB231 and LS174 cells were not affected. Indeed, P01 affected the adhesion of U87 cells on Fg and Fn with IC_50_ values of 48.86 μg/mL (15.3 µM) and 62.04 μg/mL (19.48 µM), respectively.

When compared to KAaH1, an isoform of KAaH2, active on Kv1.1 and Kv1.3 potassium channels, P01 showed a roughly comparable effect on U87 cell adhesion. Indeed, KAaH1 displayed IC_50_ values of 9.5 μM and 15 μM on Fg and Fn, respectively (Aissaoui et al., 2018). Besides, contrary to P01, at 2 h of adhesion, KAaH1 has an effect on the Pl and not with the anti-integrinic antibodies. The P01 effect on Pl significantly appeared when cells were treated for 24 h, showing that non-integrinic receptors could also be involved in the U87 cell adhesion.

For the cell migration, P01 exerted its effect on only U87 cells with an IC_50_ of 50 μg/mL (15.7 µM), while KAaH1 was active on the 3 cell lines (Aissaoui et al., 2018). Thus, P01 and KAaH1 inhibited the U87 cell migration with different mechanisms. These results brought to extent the list of substances from animal venomous reported to exhibit inhibition effects on U87 cells development, especially those from snake venoms, with other mechanism of action. For instance, PIVL, a Kunitz-type serine protease inhibitor, from the venom of the Tunisian snake *Macrovipera lebetina transmediterranea,* reduced the U87 cell adhesion, migration and invasion in the nanomoral range, by impairing the function of αvβ3 integrin (Morjen et al., 2013). Also the contortrostatin a disintegrin, that inhibited the tumor (induced by the stereotactically injection of U87 cells) progression, and prolongation of survival in a rodent glioma model (Pyrko et al., 2005). This protein specifically binds to certain integrins on the tumor cell and angiogenic endothelial cell surface and inhibits their interaction with the extracellular matrix, resulting in blockage of cell motility and invasiveness (Schmitmeier et al., 2005). On the other hand the CC-LAAO, an L-Amino Acid Oxidase from Cerastes cerastes Snake Venom, induced a dose-dependent apoptotic effect through the H_2_O_2_ generated during the enzymatic reaction (Abdelkafi-Koubaa et al., 2021).

Since P01 is highly active on SK2 channels and neither SK1 nor SK3 channels are expressed on U87 cells, we can suggest that SK2 channels are involved in the U87 cell tumorigenesis. This is in agreement with a recent paper demonstrating that SK2 channel, expressed in pancreatic ductal adenocarcinoma, increased invasiveness and metastasis formation, an effect that depends on cancer-associated fibroblasts (CAF) promoting SK2 phosphorylation through an integrin–epidermal growth factor receptor (EGFR)–AKT (Protein kinase B) axis (Rapetti-Mauss et al., 2022).

In addition, in our study, we found, in one hand, that the level of SK2 mRNA in the three cancer cells, was significantly higher than those of SK1 and SK3 (Figures 5A, B), which is in accordance with the study of (Abdullaev et al., 2010). On the other hand, we showed that the expression pattern of the SK2 isoforms was cell-type specific (Figure 5C). Indeed, while the three cancer cells express differently two isoforms at high molecular weight, only U87 cells express four SK2 isoforms at medium and smaller molecular weights. This result is in agreement with that of Strassmaier and its collaborators that highlighted the distribution and expression of four distinct SK2 channel isoforms in the human brain: the standard, the long and two short isoforms (Strassmaier et al., 2005). The shown expression of human SK2 isoforms in the brain could explain the variability of electrophysiological findings observed with SK2 channels (Willis et al., 2017).

Thus, based on the pharmacological results of P01, the specific antitumor effect of this latter on U87 cells, could be due to its interaction with these four isoforms expressed only in U87 cells, suggesting that their expression could rely on their functional activity, and their involvement in the U87 tumor mechanism.

Besides, it seems that the effect of P01 on U87 cell proliferation is not due only to its blocking activity of SK2 channels, but also by targeting a complex of membrane receptors and/or effectors expressed in U87 cells, including SK2. Indeed the use of the UCL1848 trifluoroacetate salt, which blocked SK2 channel expressed in HEK 293 cells with IC_50_ value of 0.12 nM (Hosseini et al., 2001), had no effect on U87 cell proliferation (Abdullaev et al., 2010). These authors noted that the concentrations necessary for blocking proliferation were higher than those necessary for blocking these ion channels, as we found in our study (Abdullaev et al., 2010; Abdallah et al., 2023). This suggests that the P01 and UCL1848 interact with different sites, generating different activity of SK2 channel and that the U87 cell proliferation needs the recruitment of other protein membranes to form an active complex.

Adhesion assays using blocking antibodies raised against some integrins known to be expressed in U87 and having affinity for Fg and Fn (Zhu et al., 2002; Morjen et al., 2013) indicated that P01 exerted its anti-adhesive effect by interacting with α1β1, αvβ3, α5 integrins. Thus, the specific effect of P01 on U87 cells could be due to the regulation of these integrins activity through a cross talk with the SK2 channel on the cell surface. In fact, it has been reported that K^+^ channels can interact with integrins (especially with the β subunit) and constitute macromolecular complexes associated with different cell responses (Vira and Howard, 2002; Crottès et al., 2013; Becchetti et al., 2019). Indeed, the interaction between β3 integrin and SKCa channels has been reported to play an important role in the extra-telencephalic pyramidal neurons of layer V of the medial prefrontal cortex (mPFC) of KO mice, and that the ablation of this integrin leads to an alteration of their functional activity (Vitale Carmela Thesis, 2018). Particularly, in prostate cancer, the KCa/αvβ3 integrin complex stimulates cell proliferation (Du et al., 2016). More still, in other cases, complexes can be formed by three elements (channel-integrin-receptor). For instance, in acute myeloid leukemia (AML), the Kv11.1 (hERG1) associates with β1 integrin and the vascular endothelial growth factor (VEGF) receptor 1 (also known as Flt-1), to form a complex that modulates the cell proliferation and trans-endothelial migration signaling pathways (Pillozzi et al., 2007).

## 5 Conclusion

In the light of these results, P01 is obviously a potent and specific SK2 channel blocker that may be considered a promising tool to study and target this channel in many diseases, in which it is overexpressed. Interestingly, the present study advocated the implication of SK2 channel isoforms as well as α1β1, αvβ3, α5 integrins in the selective activity of the scorpion toxin P01 against U87 glioblastoma cells.

However, to gain more insight into the mechanisms of P01 activity, further investigations are needed, especially to decipher the interaction between the SK2 channel and these integrins. Thus, this study highlights the potential of this peptide for the development of a new generation of anti-glioblastoma drugs and opens perspectives for more effective treatment strategies to handle these malignancies more successfully.

## Data Availability

The original contributions presented in the study are included in the article/supplementary materials, further inquiries can be directed to the corresponding author.

## Abbreviations

Aa, *Androctonus australis*; CH3CN, acetonitrile; Coll-I, collagen-I; DMEM, dulbecco’s modified eagle medium; DMSO, dimethylsulfoxyde; ECM, extracellular matrix; Fg, fibrinogen; Fn, fibornectin; FPLC, fast protein liquid chromatography; HPLC, high performance liquid chromatography; Lam, laminin; MTT. 3-(4, 5-dimethylthiazol-2-yl)-2,5-diphenyltetrazolium bromide; NR, Non retained AaG50 fraction; PBS, phosphate-buffered saline; PBS, phosphate-buffered saline; Pl, poly-L-lysine; SK_Ca_, small conductance calcium-activated potassium channels; TFA, trifluoroacetic acid.
